# PubTerm: a web tool for organizing, annotating and curating genes, diseases, molecules and other concepts from PubMed records

**DOI:** 10.1093/database/bay137

**Published:** 2019-01-08

**Authors:** José Garcia-Pelaez, David Rodriguez, Roberto Medina-Molina, Gerardo Garcia-Rivas, Carlos Jerjes-Sánchez, Victor Trevino

**Affiliations:** 1 Tecnologico de Monterrey, Escuela de Medicina y Ciencias de la Salud. Ave. Morones Prieto 3000, Monterrey, N.L., México; 2 Centro de Investigación Biomédica, Hospital Zambrano-Hellion, Tec Salud, Tecnologico de Monterrey, Batallón San Patricio 112 Col. Real de San Agustín, San Pedro Garza García, N.L., México

## Abstract

**Background and objective:**

Analysis, annotation and curation of biomedical scientific literature is a recurrent task in biomedical research, database curation and clinics. Commonly, the reading is centered on concepts such as genes, diseases or molecules. Database curators may also need to annotate published abstracts related to a specific topic. However, few free and intuitive tools exist to assist users in this context. Therefore, we developed PubTerm, a web tool to organize, categorize, curate and annotate a large number of PubMed abstracts related to biological entities such as genes, diseases, chemicals, species, sequence variants and other related information.

**Methods:**

A variety of interfaces were implemented to facilitate curation and annotation, including the organization of abstracts by terms, by the co-occurrence of terms or by specific phrases. Information includes statistics on the occurrence of terms. The abstracts, terms and other related information can be annotated and categorized using user-defined categories. The session information can be saved and restored, and the data can be exported to other formats.

**Results:**

The pipeline in PubTerm starts by specifying a PubMed query or list of PubMed identifiers. Then, the user can specify three lists of categories and specify what information will be highlighted in which colors. The user then utilizes the `term view’ to organize the abstracts by gene, disease, species or other information to facilitate the annotation and categorization of terms or abstracts. Other views also facilitate the exploration of abstracts and connections between terms. We have used PubTerm to quickly and efficiently curate collections of more than 400 abstracts that mention more than 350 genes to generate revised lists of susceptibility genes for diseases. An example is provided for pulmonary arterial hypertension.

**Conclusions:**

PubTerm saves time for literature revision by assisting with annotation organization and knowledge acquisition.

## Introduction

Many tasks in biomedical research require reading a large number of studies. Biocurators, for example, need to read and curate articles to feed biological or knowledge databases (1, 2). Moreover, many of the biological research project teams and postgraduate students start their research with an exhaustive analysis of published articles surrounding a certain topic. This is commonly achieved using PubMed, one of the most widely used biological literature databases (3). Nevertheless, the accumulation of scientific articles is growing at high rates, generating problems of complex organization and annotations (3). Due to the nature of biological research, the core of many projects is significantly related to well-cataloged biological entities such as diseases, genes, drugs and species. This trend has been used to automatically annotate PubMed abstracts and generate novel methods to explore biological literature (4). For example, PubTator was designed specifically to enrich the reading by highlighting specific biological terms, such as diseases, genes, drugs and species, whose annotation is available for general use (4).

There are general annotation tools like Hypothes.is (https://web.hypothes.is/) that are able to annotate any web page. Unfortunately, because of this generality, they do not provide facilities specific to biomedical research such as links to common research databases and term annotations; therefore, they are not well suited for specific curation processes. In the context of biomedical text analysis, a review of 24 alternative tools for browsing PubMed (3, 5) also highlights the fact that searching, retrieval and analysis of PubMed records is an important issue in biomedicine and research. Of these tools reviewed, there are proposals for visualization in networks [HubMed (6), RefMed (7), PubNet (8), KNALIJ (9)], searching in different ways or modalities [ask MEDLINE (10), Quertle (9), iPubMed (11), PMinstant (3), Allie (12), BabelMeSH (13), Biblimed (14), Biotext (15), GoPubMed (16), PICO (13)], expert finding [Anne O’Tate (17), eTBLAST (18), GoPubMed (16), PubFocus (19), MEDSUM (20)], identifying similar publications [eTBLAST (18), Arrowsmith (21), Dejavu (22)] or even creating models to annotate abstracts for novel concepts [ezTag (23)].

However, the curation process performed by researchers and students in general is not limited to listing all abstracts. It also involves other complex tasks such as taking notes, project-specific annotation of biological terms and abstracts and diverse ways of organizing the results. In summary, most tools focus on browsing rather than annotation, note-taking and classification.

We propose PubTerm, a web tool designed to search, acquire, categorize and annotate a list of PubMed abstracts and their biological entities (e.g. diseases, drugs, genes, species and other terms) with a design tailored toward the needs of researchers, curators and students. The pipeline starts with a PubMed query or a list of PubMed identifiers (PMID list), which after retrieval from PubMed within PubTerm, can be navigated in four views (i.e. by lists, entities, entities co-occurrence or phrases). Also, the biological entities are highlighted for easy tracking. The software is operated within common web browsers, and the work session can be saved and retrieved. The tables and annotations can be sorted, filtered and exported for further use. We provide details of all interfaces in the tool’s tutorial. As an example, we successfully used PubTerm to generate a revised list of susceptibility genes involved in pulmonary arterial hypertension (PAH) (24).

## Computational methods

During the development of PubTerm, we observed that there are functionalities implemented in other tools that can be beneficial for an annotation and organization tool. In addition, through several cycles of design, deployment and testing, we collected requirements for accomplishing the necessary annotations and curations of the motivational example (gene panel design). In summary, these are described next.

***Friendly abstract retrieval*.** We observed that other tools implement specific methods to find PubMed records. This is counterintuitive since the user must first learn the query method. Therefore, we used the PubMed API to query PubMed records in exactly the same way that the user is already used to doing on the PubMed web page. By doing this, the user is able to first query PubMed in the usual way, and once it yields the desired results, the query can be copied and pasted into PubTerm. Unfortunately, with this method, the PubMed API only allows for the retrieval of 1000 records. Thus, we additionally implemented the second method in which the PMIDs can be pasted to process more than 1000 records at once.

***Term highlighting*.** The identification and highlighting of key concepts or `terms’ such as genes, species, chemicals or drugs and diseases are of special value for the biomedical community. This facility saves time, allowing the user to focus their reading on specific sections of the PubMed abstract. For this, we used the `PubTator’ annotations (4), which are added to the PubMed record and highlighted when displaying the abstract text. For highlighting, we additionally added configurable colors and switches to determine whether specific terms should be highlighted or not. PubTator data is updated monthly in our servers.

***Annotation*.** Most of the reviewed tools (3, 5) do not allow for writing notes on records. We have found that this is the main need for many users who want to write notes associated with abstracts. For instance, if an abstract is of special value, they may want to add, `The authors show for the first time that …’. Therefore, we designed PubTerm so that notes can be associated with abstracts, but also with all other terms, such as genes, diseases, species, drugs or any other term included (see `Organization by term’ below).

***Categorization*.** In addition to annotations, we allow the user to define three categories to document their assessment of the abstract or terms (e.g. useful/not useful, strong evidence/weak evidence/no evidence).

***Marking*.** Besides annotation and categorization, the text within abstracts can marked to highlight remarks.

***Organization by term*.** Some tools like CoolGen (25) (http://ci.smu.edu.cn/CooLGeN) organize abstracts only by genes. Inspired by this feature, we designed PubTerm to organize abstracts by other terms as well. The terms include not only those from PubTator (Genes, Diseases, Chemicals, Species, Mutations/Variants), but also user-defined categories, journal, first or any authors, affiliation, year and year and month. The fields shown within the generated term table can be configurable, which includes terms, ID, species, categories, statistics and notes.

***Other views*.** Besides organization of abstracts by terms (`term view’), we also added another three views that may aid annotation and exploration. The `records view’ shows the list of all abstracts, where notes, categories and marks can be edited. The fields shown in the table are configurable. The table can be sorted by any field and filtered by any text. In addition, abstracts can be manually deactivated from analysis. The *`*co-occurrence view’ shows a matrix of terms where the intersection shows the number of abstracts that mention or refer to both terms, which can then be viewed and edited. This view is useful for exploring the dependency of certain terms, for example, genes and diseases. The `sentence view’ is designed to show the context of words within abstracts. It shows a number of characters before and after specified words. The abstracts referred to can be viewed and edited.

***Friendly results viewing and filtering*.** Many of the results are shown in tables. Thus, to improve functionalities, we used the DataTables plug-in (https://datatables.net) for jQuery (https://jquery.com). By taking advantage of available features, all results shown as DataTables can be easily sorted, filtered, browsed, copied, exported and printed.

***Computational platform independence*.** To improve the functionalities, we used software packages compatible with most internet browsers. We preferred internet browsers rather than native applications to avoid complexities derived from the diversity of operating systems. For this, we used HTML5, JavaScript, jQuery and DataTables.

***Saving and retrieving annotations*.** All user notes, marks, categories and other configuration settings can be saved in our servers by specifying an e-mail address and project name. Changes can be semi-automatically saved and even reviewed, which is helpful for rolling back changes.

***Term statistics*.** Because abstracts can be organized by terms, which are mentioned at different frequencies, a recurrent question within the curation processes is whether the observed term frequency is due to random chance. Therefore, we estimated some indicators that can help in this context. In particular, we estimated the number of times a term seems to be over-represented (folds) within the collection of abstracts (observed ratio) relative to the whole abstract database (theoretical ratio). In addition, we estimated the probability of observing the frequency of the term by chance via a hypergeometric test followed by a correction for multiple tests using a false discovery rate approach (26).

***Abstract record.*** Because the abstract is the most important reading unit, we provided a set of tools to facilitate curation. Genes, diseases, chemicals, species and sequence variants can be clicked on to open the PubMed record or to open the PubTerm record for viewing and editing. Authors, affiliations and journals can also be clicked on for general searches.

## System description

**Figure 1:**
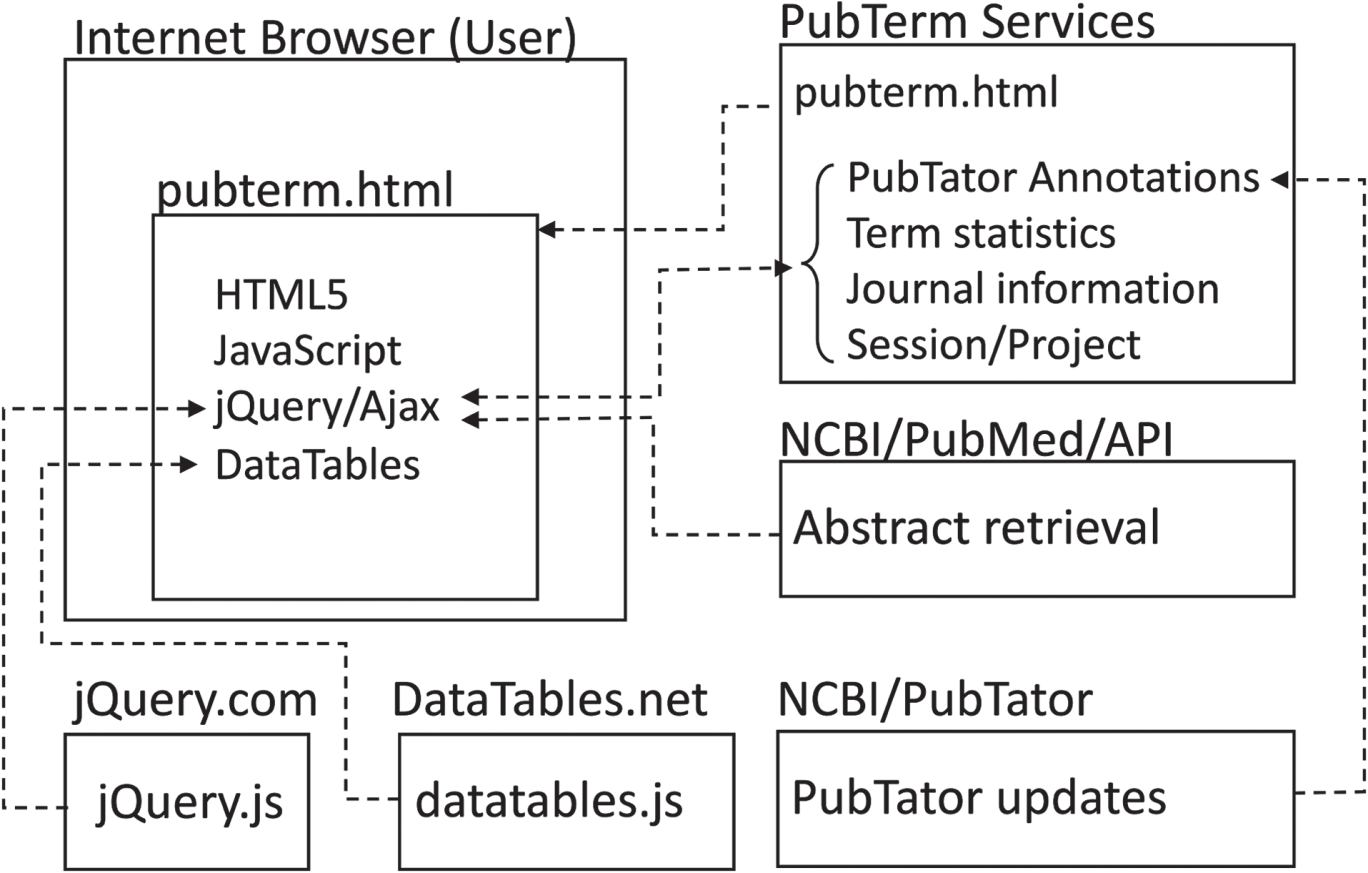
Implementation of PubTerm. Each box represents a server for computational services or a user browser. Dashed lines represent requests/responses and arrowheads represent flows of data.

A summary of the PubTerm implementation is shown in Figure 1. As can be observed, PubTerm is a lightweight web application running in the user’s browser. The servers shown provide the information, which basically consists of abstract records, pre-computed data and software tools. The PubTerm services were implemented as JavaServer Pages web services.

**Figure 2:**
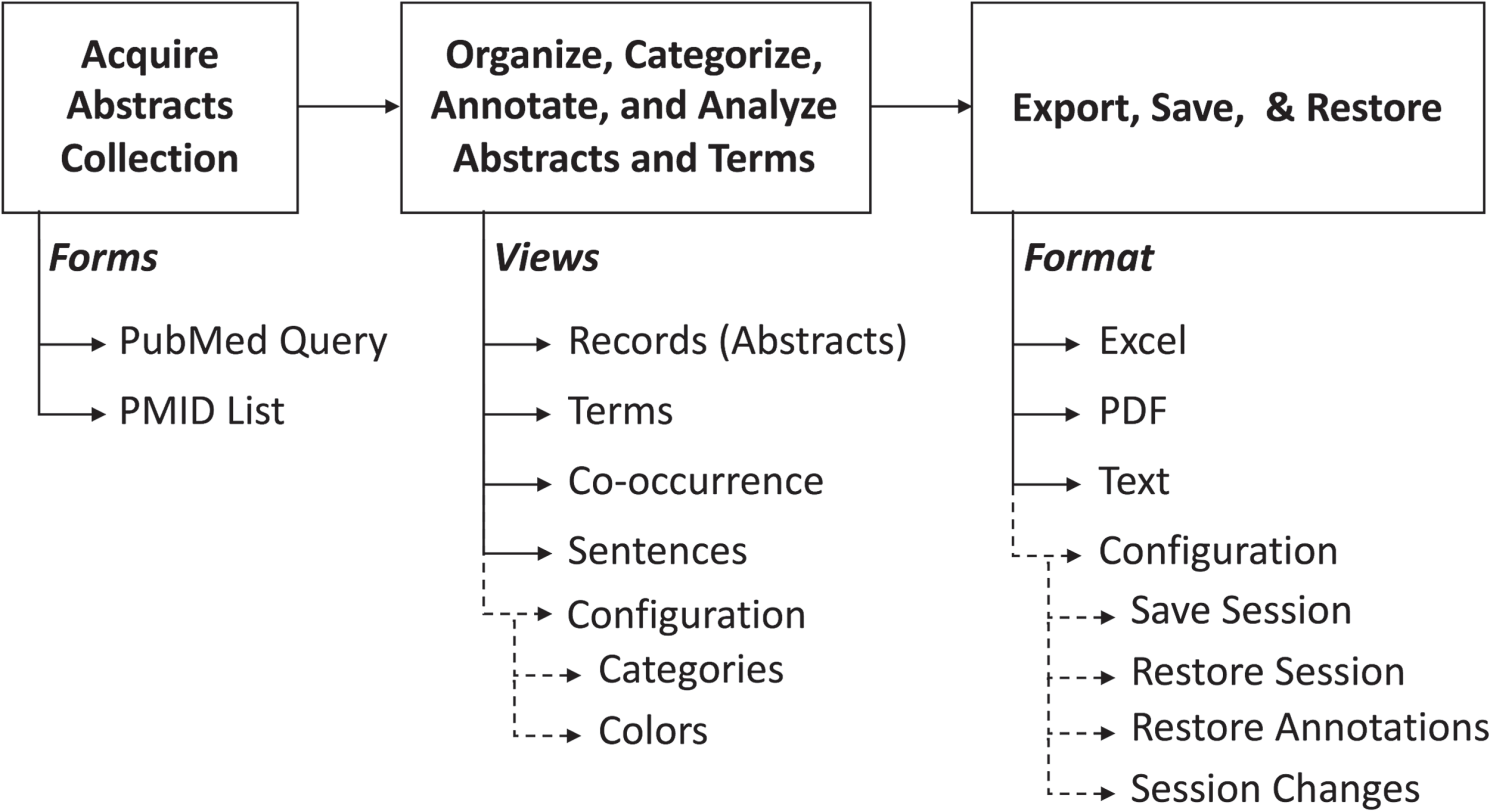
Summary of PubTerm. The top scheme shows the typical pipeline. Below, the forms, views and options are shown.

The common operational process is shown in Figure 2. Briefly, it is composed of (i) input, (ii) annotation, categorization, curation and analysis and (iii) saving and restoring the session data. The following paragraphs will provide details about each stage.

## Input forms

**Figure 3:**
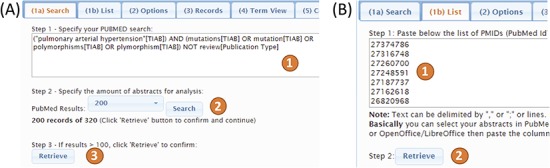
Input methods for PubTerm. (A) Using a PubMed query in (1), specifying the number of records (2) and loading them up (3). (B) Using a list of PubMed IDs in (1) then loading them in (2).

PubTerm needs a list of PubMed records. To procure this, it accepts query words in exactly the same way as PubMed, which are then sent to the PubMed API to retrieve the list of records (Figure 3A). Up to 1000 records can be retrieved using this method due to PubMed API restrictions. The time needed to load the abstracts is typically less than 1 min. The second method is designed to load more than 1000 records. For this, the user needs to feed PubTerm the precise list of PMIDs (Figure 3B). This list can be easily obtained from PubMed exports. For example, using the option `sent to-> file’ within the PubMed results will produce the list (see the `Help/Tutorial’ section for details).

## Configuration options for organization and text highlighting

We used PubTator to annotate diseases, genes, drugs, species and mutations/variants (4) as a general mechanism to organize and highlight information from the abstracts. Colors can be configured or deactivated. The user can configure three categories containing a customized list of values, which can be used to classify abstracts or biological entities (e.g. genes, diseases or drugs). The text within an abstract can also be marked, and several links are provided for terms, authors, journals, affiliations and other information.

## Annotations and categorizations

**Figure 4:**
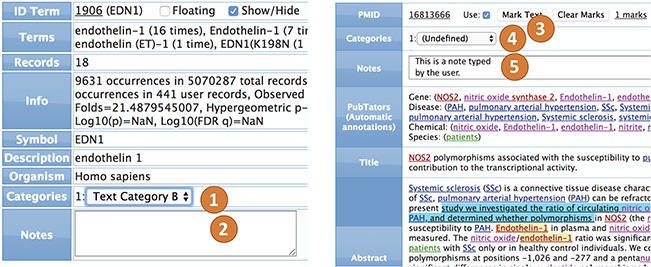
Annotation and categorization. The terms (left) can be annotated by categories (1) and notes (2). The text within abstracts (right) can be marked (3), but the abstract itself can also be categorized (4) and annotated (5).

***Terms.*** All terms (genes, diseases, chemicals, species, sequence variants, user categories and abstract fields) can be annotated and categorized (Figure 4). For example, in a collection of 50 abstracts in which there are 20 genes involved, each of the 20 genes can be annotated and categorized. The annotation is a free text that keeps a record of user notes. The categories consist of lists of user texts that can be configured (see `Options’). The values of each category can then be selected during term editing. The most common way to edit annotations and categories for a term is by using the `term view’.

***Abstracts.*** All interfaces can interact with abstract records, which are shown similarly to PubMed and PubTator, but they can also show and edit user notes, categories and marks (Figure 4). Most of the recognized information can be linked to original records in PubMed, NCBI databases or on the internet. The user can also view and edit the annotations of the terms within the abstract interface.

## Views

**Figure 5:**
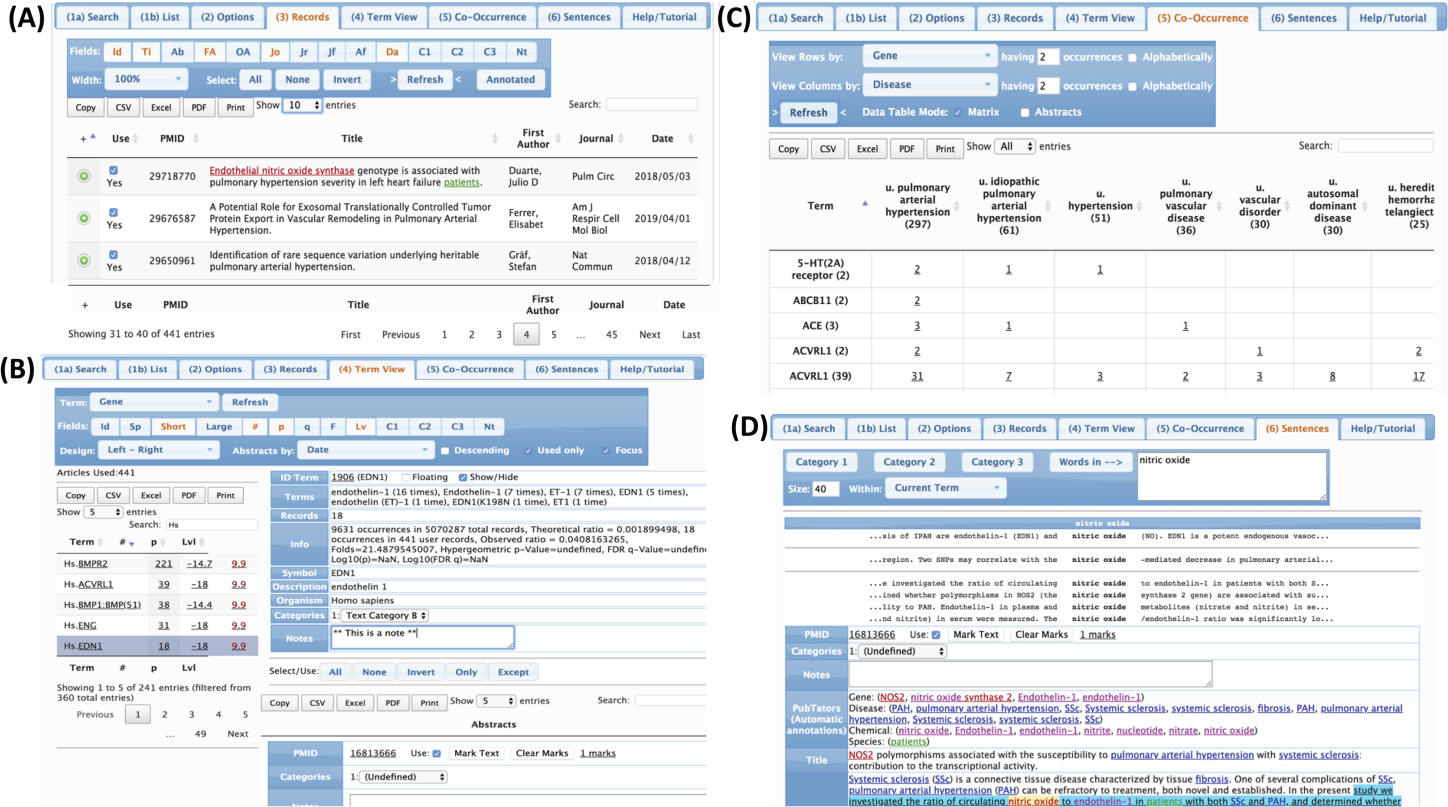
Views of the abstracts and terms. (A) Record view. (B) Term view. (C) Co-occurrence view. (D) Sentence view.

PubTerm implements four views to navigate and organize abstracts, terms and annotations (Figure 5), which are described below.

***Records view.*** This interface shows the list of abstracts. The user can choose abstract fields to show, check how the list is sorted, and filter records by keywords. From the list, the user can select any abstract and edit the annotations, categories and marks.

***Term view.*** This is the main interface, and it organizes the abstracts by terms. The terms can be genes, diseases, chemicals, species, sequence variants (mutations) or customized user categories, as well as other abstract information such as the journal, author, affiliations and year. After selecting the term and possible fields, PubTerm shows a list of all values found for that term together with some statistics, such as the number of abstracts per term and a level of non-random assignment (see the `Tutorial’). For example, if the user selects the term `Gene’, PubTerm lists all the genes mentioned in the abstracts ordered by the number of occurrences (the list can also be easily sorted and filtered). If the user selects a gene within the list, all abstracts mentioning that gene will be shown as a list. The user can then annotate the term and/or any of the associated abstracts.

***Co-occurrence view.*** This interface is designed to analyze possible associations between terms. For example, when examining genes and diseases, a matrix of all genes and diseases is formed, providing the number of abstracts in which both terms are mentioned, which may help to determine where the gene has major reported effects. Moreover, the co-occurring abstracts can be listed for reviewing and editing. This may help to reveal useful associations needed for the annotation or curation process.

***Sentence view.*** In this view, the user can explore all sentences within abstracts that contain specific words in order to analyze the context in which the words are used. The user can then open the abstract related to the sentences for editing.

## Saving and exporting

***Exporting.*** In all views, the results can be exported to Excel, PDF, Text or Printer. This allows for further processing and analysis.

***Saving and restoring sessions.*** All annotations, categorizations and marks can be saved within the PubTerm server. For this, the user’s e-mail address and a project name are needed; secret keywords can optionally be added. Once saved, the user can retrieve all annotations at any time using the user and project information. Annotations can also be shared between projects using the `Restore Annotations’ option. In addition, PubTerm keeps a record of changes made in the current session to save them easily, to assist with revisions, and, manually, to roll back changes.

## Help and tutorial

PubTerm includes a brief description of features within the main interface and instructions for the correct setup. It also links to a more detailed tutorial that explains the inputs and outputs and provides step-by-step examples of the use of PubTerm.

## Hardware and software specifications

PubTerm can be operated in any current internet browser regardless of the operating system. PubTerm uses standard HTML5 and JavaScript capabilities. It also requires jQuery and DataTables, which are loaded as needed with no user configuration. Nevertheless, because PubTerm performs requests to third-party domains such as NCBI, it may need the installation and `configuration of cross-origin resource sharing’ that allows browsers to make requests to different domains. In Chrome, we have used the add-on Allow-Control-Allow-Origin v1.0.3. Firefox v60.0 does not seem to need plug-ins, but older versions may need the CorsE plug-in. In Safari, PubTerm needs to disable cross-origin restrictions available within the `Develop’ menu. Other browsers compatible with HTML5 and JavaScript should work if cross-origin is configured properly.

## Example of typical operation

As a conceptual demonstration of PubTerm’s capabilities, we will briefly describe the operations used to define a gene panel for the PAH disease (24). In this example, we focus on annotating and categorizing genes only, but PubTerm is not limited to genes. Full details are provided within the tutorial available in PubTerm under the `Help/Tutorial’ section or at http://bioinformatica.mty.itesm.mx/PubTerm. The original goal of the demonstration was to determine the set of genes having published evidence of susceptibility to PAH.

## Specifying inputs

After some trials on the PubMed main page, we used the following query in PubTerm (input form 1a): `(((pulmonary[TIAB] AND arterial[TIAB] AND hypertension[TIAB]) AND (mutations[TIAB] OR mutation[TIAB] OR polymorphisms[TIAB] OR polymorphism[TIAB] OR allele[TIAB] OR alleles[TIAB] OR SNP[TIAB] OR ``copy number”[TIAB] OR ``aberration”[TIAB]))) NOT Review[Publication Type]’. This query will retrieve PubMed abstracts that are related to PAH and refer to gene polymorphisms, avoiding review publications for this particular task. After using the buttons `Search’ and `Retrieve’, PubTerm loaded more than 400 abstracts from PubMed.

## Configuration of the project

The task was determining which genes were truly associated with PAH. Therefore, to facilitate notes, we used the user-defined categories from PubTerm to define the following values in Category 1: (i) `Experimental evidence of mutations’, (ii) `Other genetic evidence’, (iii) `Related but not mutated’, (iv) `Unrelated’, (5) `Annotation error’, (6) `Negative evidence of mutations’ and (7) `Genetic alteration in related disease’. Then, one of these values was assigned to each gene as detailed below.

## Curation, categorization and making notes

We mainly used the `term view’ from PubTerm to curate abstracts. Overall, the more than 400 abstracts mentioned around 371 genes, which are by default listed with the number of abstracts mentioning that gene and a confidence level representing the likelihood of observing that gene by chance (higher values indicate more confidence that observations are not due to chance). Nevertheless, from the 371 genes, only 229 were annotated as human genes. This was easily found by typing `Hs.’ (meaning *Homo sapiens*) in the filtering box of the gene table. Then, we clicked the first gene to list all abstracts mentioning that gene. The first gene was BMPR2, which is well known to be associated with PAH. The BMPR2 gene was mentioned by 181 of the 405 abstracts, having a confidence level of 9.9 (the maximum). The statistics provided show that BMPR2 is mentioned 1195 times in more than 5 million PubMed records that were annotated. Thus, 181 mentions in 405 abstracts represent a frequency around 1900 times higher than expected. Therefore, it is unlikely that BMPR2 is not associated with PAH. Nevertheless, the curation process and protocol definition required further reading, marking the sentences of evidence and taking notes. Therefore, after reading a few abstracts and marking evidence, it was clear that BMPR2 should be defined as `Experimental evidence of mutations’, specifying this in the list of options related to Category 1. In this way, the curation process involved reading only a few abstracts rather than the 181 referring to BMPR2. The following genes listed were similarly revised. Many genes were even easier to examine, since the number of abstracts per gene decreased rapidly. For instance, the gene ranked 20 has only seven abstracts registered, the gene ranked 50 has three and so on. Many other genes had only one or two abstracts, which facilitates the reading and categorization process. After a few curation sessions, we defined 21 genes showing clear evidence of sequence variants and a few others with potential associations (24). Most importantly, we found that most of the 371 recognized genes were not associated, which was very difficult to define in the first place.

## Session information

The curation process was performed during several 1- or 2-h sessions in which changes were saved almost automatically once configured. We have not measured the entire process, but overall, the revision should take around 20 h (for about 300 genes). We have observed that users quickly get used to navigating the tool and make faster annotations over time. The data for this example can be accessed in PubTerm using `Options’, `Restore Session’, `e-Mail’ = vtrevino@itesm.mx, `Project’ = PAH Final-405.

## Discussion

In many research and development biomedical projects, there is a considerable amount of time spent on literature review and curation. Although a large list of software has been devoted to browsing, almost no software focuses on annotation. Here, we proposed PubTerm, a simple system to acquire, curate, annotate and categorize not only abstracts, but also genes, diseases, species, drugs, sequence variants and other journal- and author-related information. We have successfully used PubTerm to design a gene panel to genetically screen for PAH. We are using PubTerm to analyze literature on other diseases, including Crohn’s disease and sclerosis lateral amyotrophic. Because of the design that combines abstract annotations and various views, we believe that PubTerm can be used in many other biomedical tasks involving reading or analysis of many abstracts.

One of the caveats of using PubTerm is the fact that most of the information is loaded in real time from the original servers (or images of the databases in the case of PubTator annotations). In the current version, PubTerm saves the project session containing the current state of annotations, marks and categories for terms and abstracts, but not the PubMed abstract record nor its annotations. This might present some inconsistencies after long time periods when the annotations change. For example, when we originally generated the gene panel for PAH, we found a gene named PAH that was wrongly recognized as a gene instead of the disease of the same acronym. A recent version of PubTator seems to correct this issue and PAH is no longer in the list of genes. We will try to correct this issue in future versions, giving users the option to save the annotations and abstracts together with the project session.

Another point of improvement is the annotation of terms. Currently, PubTerm relies on PubTator annotations. However, novel tools recently proposed, such as ezTag (23), could, in principle and with a properly trained model, annotate all abstracts and make them available for PubTerm. This could be an interesting future direction.

We conclude that PubTerm is a valuable tool for biomedical research and development projects involving the review or curation of many abstracts.

## Notes

**Database URL**: http://bioinformatica.mty.itesm.mx:8080/Biomatec/pubterm.html
